# Leptin and EGF Supplementation Enhance the Immune System Maturation in Preterm Suckling Rats

**DOI:** 10.3390/nu11102380

**Published:** 2019-10-06

**Authors:** Blanca Grases-Pintó, Paulina Torres-Castro, Lidia Marín-Morote, Mar Abril-Gil, Margarida Castell, María J. Rodríguez-Lagunas, Francisco J. Pérez-Cano, Àngels Franch

**Affiliations:** 1 Physiology Section, Department of Biochemistry and Physiology, Faculty of Pharmacy and Food Science, University of Barcelona, 08028 Barcelona, Spain; blancagrases@ub.edu (B.G.-P.); mtorreca29@alumnes.ub.edu (P.T.-C.); lmarinmorote@gmail.com (L.M.-M.); mariadelmar.abril@ub.edu (M.A.-G.); mjrodriguez@ub.edu (M.J.R.-L.); angelsfranch@ub.edu (À.F.); 2 Nutrition and Food Safety Research Institute (INSA·UB), 08921 Santa Coloma de Gramenet, Spain

**Keywords:** prematurity, suckling rat, leptin, EGF, adaptive immunity, innate immunity, breast milk

## Abstract

In preterm newborns the immaturity of the immune system is remarkable, with reduced innate and adaptive immune responses. Many bioactive compounds in breast milk, such as growth factors and adipokines, contribute to the immune system’s maturation in early life. However, studies on the immunoregulatory activity in preterm neonates are practically nonexistent. The aim of the present study was to determine whether a nutritional supplementation in early life with leptin or epidermal growth factor (EGF) was able to promote the maturation of the systemic and intestinal immune system in preterm conditions. For this purpose, premature rats were daily supplemented by oral gavage with leptin or EGF. Term and Preterm groups receiving vehicle were used as controls. Preterm rats showed deficiencies compared to full-term ones, such as lower body weights, erythrocyte counts, plasma IgG and IgM concentrations and B cell percentages, and higher values of Th and Tc TCRαβ^+^ cells in mesenteric lymph nodes, and intestinal permeability, among others. However, leptin and EGF supplementation were able to revert some of these deficiencies and to improve the premature immune system’s development. These results suggest that leptin and EGF are involved in enhancing the maturation of the systemic and intestinal immune system in preterm conditions.

## 1. Introduction

Prematurity is one of the main causes of neonatal death. Advances in medicine over the years have enabled the survival of preterm newborns who would previously have died [1]. In premature infants, the third trimester is missed, triggering an incomplete development in the fetus and its organs, making these babies more susceptible to neural and respiratory problems, among other complications [2]. Moreover, the gastrointestinal tract of term infants—which should also develop in the last trimester—is immature at the time of birth, and consequently, more undeveloped in preterm newborns than in term ones. The underdeveloped intestine and other factors, such as the immature intestinal immune system, unestablished microbiota, or the high intestinal permeability, make the premature infant predisposed to suffering from necrotizing enterocolitis (NEC), a common disease in premature babies [3]. 

It is well known that breast milk constitutes the optimal source of nutrition for the newborn [4]. As this is also the first choice to nourish preterm babies, if the mother is unable to produce the volume of milk required, donor milk is preferred to infant formula [5]. However, in cases where the newborn is very premature, parenteral nutrition is the first option, since the intestine is not ready to receive food [6]. Nevertheless, 5 days after birth the mother can start to give small volumes of breast milk until reaching the enteral volume, reducing parenteral nutrition and facilitating weight gain [7]; and when the preterm infants’ birth weight is very low, it is recommended to supplement the donor milk with fortifier powders from bovine milk to increase protein, calcium, and vitamin D levels, among other factors [8]. Hence, breast milk feeding is recommended for preterm babies because of its effect of reducing NEC [9,10,11,12], promoting brain development [13], stimulating intestinal maturation [6], promoting the development of oral tolerance [14], and reducing infections [15]. Moreover, preterm breastfed infants showed a lower metabolic syndrome rate, less insulin and leptin resistance, and lower blood pressure, later in life than newborns nourished with infant formula [5,16]. 

In addition to inter and intra-individual differences among the milk composition of mothers, some differences between term and preterm milk have also been described. Hence, preterm breast milk has higher levels of protein, free amino acids, and sodium [5], but lower levels of lactose have been described [17]. Interestingly, preterm breast milk has higher concentrations of immune factors such as lactoferrin, cytokines (IL-6, IL-10, TNF-α), and secretory immunoglobulin (Ig)A than term breast milk [5], stimulating the immature immune system of the preterm newborn [18]. In addition, breast milk contains other bioactive factors with immunomodulatory properties, such as growth factors (transforming growth factor (TGF)-β and epidermal growth factor (EGF)) and adipokines (leptin and adiponectin) [19,20]. We have recently demonstrated in term rats that leptin and EGF supplementations during the suckling period are able to enhance early immune system development [21,22,23]. However, the influence of these bioactive compounds on the preterm immune system remains unexplored. We hypothesize that these milk bioactive factors could also have a role in the development of the immature immune system in premature conditions. Thus, the aim of the present study was to determine the effects of supplementation with leptin and EGF on the immune systems of premature suckling rats. Specifically, we focused the study on evaluating the effects of leptin and EGF on different variables of innate and adaptive immunity affected by premature delivery [24].

## 2. Materials and Methods 

### 2.1. Animals

Twenty-one pregnant Wistar rats from Janvier Labs (Le Genest-Saint-Isle, France) at different stages of gestation were used. Specifically, nine of them were at 13 days of gestation (G13), three at 14 days of gestation (G14) and nine at 15 days of gestation (G15). Dams were individually housed in cages, fed with chow and water *ad libitum*, and monitored daily. The animals were maintained under controlled temperature and humidity conditions, in a 12:12 h light:dark cycle in the Animal Facility of the Faculty of Pharmacy and Food Science. 

The studies were performed in accordance with the criteria outlined by the Guide for the Care and Use of Laboratory Animals. Experimental procedures were reviewed and approved by the Ethical Committee for Animal Experimentation of the University of Barcelona (CEEA/UB reference 148/18).

### 2.2. Experimental Design

Three G14 pregnant dams were allowed to deliver naturally at term (day 22 of gestation). G13 pregnant dams (*n* = 9) had a caesarean (C)-section one day before normal delivery (day 21 of gestation), giving birth to preterm pups. Due to the possible impact of surgery on the dams, they were not allowed to keep the neonates, so the pups were accepted and breastfed by surrogate dams. G15 dams (*n* = 9) were allowed to deliver at term one day before term dams. These dams nursed the pups born in preterm conditions (i.e., surrogate dams). It was important that surrogate dams had delivered two days before the C-section to ensure that they had enough milk in their breasts to breastfeed preterm pups. Litters were culled to 10 pups per lactating dam and they had free access to the nipples and rat diet during 17 days of the suckling period. Handling was done in the same time range to avoid the influence of biological rhythms.

### 2.3. Dietary Supplementation

Rats were distributed into four experimental groups: Term (T), Preterm (P), P+Leptin and P+EGF. Each group was composed of three litters (*n* = 30 pups/group). T group was formed by the three lactating dams with their corresponding litters delivered at term. P group was formed by three surrogate mothers with three litters delivered by C-section. P+Leptin group was formed by another three surrogate mothers with three litters delivered by C-section and supplemented during 17 days with a solution of 0.7 μg/kg/day of leptin (PeproTech^®^, Rocky Hill, NJ, USA) in mineral water. The P+EGF group was formed by another three surrogate mothers with three litters from those delivered by C-section but they were supplemented over 17 days with a solution of 100 μg/kg/day of EGF (PeproTech^®^) in mineral water. These doses were selected according to previous studies showing immunomodulatory effects [21,22,23]. The T and P groups were administered with the same volumes of vehicle as the supplemented groups (10 mL/kg/day) during the first 17 days of suckling. To allow gastric emptying, litters were separated from their dam a half-hour before oral supplementation. Meanwhile animals were weighed daily. Pups received the supplements daily by oral gavage using low-capacity syringes (Hamilton Bonaduz, Bonaduz, Switzerland) adapted to oral 25 or 23-gauge gavage tubes (ASICO, Westmont, IL, USA), as previously described [22].

On the days of sacrifice (i.e., 10 and 17), the lengths (nose−anus) of the animals were also measured. On the same days, the body mass index (BMI) was determined, calculated as body weight/length^2^ (g/cm^2^), as was the Lee index, calculated as ^3^√weight/length × 1000 (^3^√g/cm).

### 2.4. Caesarean Intervention 

To obtain premature pups in P, P+Leptin, and P+EGF groups, a C-section at G21 was required. The procedure was based on the methodology described previously [24]. Dams, anesthetized with isofluoran inhalation, were immediately sacrificed by cervical dislocation, in order to avoid pups being affected by the administration of a longer exposure to anesthesia. Immediately, the offspring were extracted one by one by hysterectomy. They were separated from the placenta, the umbilical cord was cut and emptied, the airway was cleared of fluid with a paper towel, and respiratory function was activated with a soft massage on the chest. They were then cleaned carefully with warm physiological serum (37 °C) to remove the remaining blood, and a knot was made with the umbilical cord. Then, they were randomly distributed among the nine surrogate mothers’ cages. Preterm pups were mixed with the bedding of the cage so that they acquired the smell of the new mother to avoid their rejection. Preterm rats were accepted by the corresponding surrogate mothers, which had previously been separated from their own offspring, except two pups that were kept to help prevent cannibalism by the surrogate dam.

### 2.5. Sample Collection and Processing

At day 10, animals were intramuscularly anesthetized with ketamine (90 mg/kg) (Merial Laboratories S.A., Barcelona, Spain) and xylazine (10 mg/kg) (Bayer A.G., Leverkusen, Germany), exsanguinated, and the small intestine (SI) was collected. On this day, three random rats from each litter (9 rats/group) were used to analyze blood cell count, plasmatic Ig concentration, and the phagocytic activity of leukocytes. Another three rats from each litter (9 rats/group) were used to perform histomorphometric and immunofluorescence staining studies and determine the gene expressions and permeability of the SIs. Moreover, livers, spleens, and thymuses from all rats were collected and weighed. In addition, small and large intestines were measured.

Blood samples collected in heparin/lithium tubes were used to perform the phagocytic assay. After that, the remaining blood was centrifuged to obtain plasma for Ig quantification following previous conditions [22]. An automated hematological analyzer (Spincell 3, Spinreact, Barcelona, Spain) was used to determine the composition of the cellular elements of the blood. For the histological study, the SI was washed and a 1 cm portion from the distal jejunum was cut, placed in cassettes and fixed in 4% paraformaldehyde, as previously described [24]. Moreover, another 0.5 cm portion of the distal jejunum was immediately conserved in RNAlater^®^ (Ambion, Applied Biosystems, Austin, TX, USA), incubated at 4 °C overnight, and stored at −20 °C until PCR analysis.

At day 17, rats were anesthetized as described above. Similar to day 10, blood samples were collected to determine the Ig pattern from plasma. Furthermore, livers, thymuses, and spleens were weighted, and intestines were measured. Moreover, mesenteric lymph nodes (MLNs) were also obtained [22].

### 2.6. Phagocytic Assay

To evaluate the phagocytic activity of leukocytes, the commercial kit Phagotest^®^ (Glycotope, Biotechnology, Heidelberg, Germany) was used according to the manufacturer’s instructions, as previously described [24]. Briefly, opsonized fluorescein isothiocyanate (FITC)-labelled *Escherichia coli* were added to the heparinized blood and incubated for 10 min at 37 °C. Then, the tubes were placed in ice to stop phagocytosis. After washing and centrifugation, cells were incubated in a lysis solution in order to eliminate the erythrocytes and fix the leukocytes. Finally, the cellular DNA was stained with a propidium iodide solution.

Analyses were performed using a Gallios^TM^ flow cytometer (Beckman Coulter, Miami, FL, USA) at the Scientific and Technological Centers of the University of Barcelona (CCiT-UB) and assessed by FlowJo v10 software (Tree Star Inc., Ashland, OR, USA) as previously described [24]. The phagocytic activity was expressed as the percentage of fluorescent cells (monocytes or granulocytes) in the particular population studied. The mean fluorescence intensity, indicative of the extent of phagocyte efficiency was also quantified.

### 2.7. Immunoglobulin Quantification

Plasma IgA, IgM, IgG1, IgG2a, IgG2b, and IgG2c concentrations were quantified by a ProcartaPlex Rat Antibody Isotyping Panel (eBioscience, Frankfurt, Germany), according to the manufacturer’s protocol and previous studies [22]. Results were analyzed by the Luminex MAGPIX analyzer (Luminex^®^, Austin, TX, USA) at the CCiT-UB. Assay sensitivity was as follows: 0.48 pg/mL for IgA, 0.02 ng/mL for IgM, 0.78 ng/mL for IgG1, 0.02 ng/mL for IgG2a, 0.11 ng/mL for IgG2b, and 0.19 pg/mL for IgG2c.

### 2.8. MLN and Spleen Lymphocytes Isolation

Lymphocytes from MLNs and spleens were isolated, as previously described [21,22], by passing the tissues individually through a sterile 40 µm mesh cell strainer (Thermo Fisher Scientific, Barcelona, Spain). A resultant cell suspension from the spleen required an additional step to lyse the erythrocytes [22]. Cell counting and viability were assessed by Countess^TM^ Automated Cell Counter (Invitrogen^TM^, Thermo Fisher Scientific). Lymphocytes from both organs were then used to study their phenotype.

### 2.9. Lymphocyte Immunofluorescence Staining and Flow Cytometry Analysis

For flow cytometry analysis, lymphocytes (2 × 10^5^) from spleens and MLNs were labeled with mouse anti-rat monoclonal antibodies (mAb) conjugated to FITC, phycoerythrin (PE), peridinin-chlorophyll-a protein (PercP), allophycocyanin (APC), or BD Horizon™ BV421, as in previous studies [21,22]. In this case, the mAb used were anti-CD4, anti-CD8α, anti-CD8β, anti-TCRαβ, anti-NKR-P1A, anti-TCRγδ, and anti-CD45RA (BD Biosciences, San Diego, USA). After staining with standard procedures [21], analyses were performed using a Gallios^TM^ Cytometer (Beckman Coulter, Miami, FL, USA) at the CCiT-UB. All results were assessed by the FlowJo v.10 software.

### 2.10. Intestinal Permeability Assay

At day 10, the permeability of the intestinal epithelial barrier was also determined *in vivo* by the paracellular passage of 4 kDa-dextran into the blood, as previously described [24]. Briefly, a solution of 4 kDa-dextran conjugated to FITC (Sigma-Aldrich, St. Louis, MO, USA) was orally administered to rats using the low-capacity syringes adapted to oral gavage tubes. There was an additional group of animals that was only administered with an equivalent volume of PBS (10 mL/kg) to rule out the background fluorescent levels of the types of samples. After 4 h of the dextran administration, the animals were euthanized and plasma was obtained, diluted, and the fluorescence emission was quantified in triplicate at an excitation wavelength of 490 nm in the Modulus™ Microplate spectrophotometer (Turner Byiosystems, Sunnyvale, CA, USA).

### 2.11. Periodic Acid−Schiff Staining

By increasing the gradient of ethanol, fixed intestinal samples were dehydrated. Then, they were paraffin-embedded, cut into 5 μm sections, deparaffinized and rehydrated for periodic acid−Schiff (PAS) staining, as previously described [24]. The observation of the intestinal architecture was performed using the bright-field of an Olympus BX41 microscope (Olympus Corporation, Shinjuku, Tokyo, Japan). All the morphometric measurements were processed with the ImageJ program (image processing and analysis in Java, National Institute of Mental Health, Bethesda, MD, USA). The sample size was six animals, representative of the three litters in each experimental group (2 animals/litter). Six to ten villi were selected randomly from each animal and the villi’s heights, widths and epithelium perimeters were measured. Moreover, the number of goblet cells per villus and their corresponding areas were also evaluated.

### 2.12. Small Intestine Gene Expression

SI portions kept in RNAlater^®^ were homogenized for 30 s in lysing matrix tubes (MP Biomedicals, Illkirch, France) using a FastPrep-24 instrument (MP Biomedicals), as previously described [25]. Total RNA was extracted by RNeasy^®^ mini kit (Qiagen, Madrid, Spain) following the manufacturer’s instructions. RNA quantification was performed with a NanoPhotometer (BioNova Scientific, CA, USA). Later, cDNA was obtained in a thermal cycler PTC-100 Programmable Thermal Controller using TaqMan^®^ Reverse Transcription Reagents (Applied Biosystems, Weiterstadt, Germany). The specific PCR TaqMan^®^ primers (Applied Biosystems) used to assess gene expression with real-time PCR (ABI Prism 7900 HT, AB) were: *MUC-2* (Rn01498206_m1), *MUC-3* (Rn01481134_m1), *Prdm1* (Rn03416161_m1, I, encoding for Blimp-1), *Fcgrt* (Rn00583712_m1, I, encoding for FcRn), *zona occludens (ZO)-1* (Rn02116071_s1), *occludin* (Rn00580064_m1), and *claudin-4* (Rn01196224_s1). The relative gene expressions were normalized with the housekeeping gene Gusb (Rn00566655_m1, I) using the 2–∆∆Ct method, as previously described [26]. Results are expressed as percentage of values of each supplemented group normalized to the mean value obtained for the reference group (T group), which was set at 100%.

### 2.13. Immunofluorescence Study of Tight-Junction Proteins

Immunofluorescence staining for occludin, ZO-1, claudin-2, and claudin-4 proteins were performed using the same paraffin-embedded intestine previously described for PAS staining. Briefly, after deparaffinizing the slides with xylene (Honeywell Chemicals, Diegem, Belgium) for 25 min at 60 °C, the intestine sections were rehydrated in serial dilutions of 100%–30% ethanol, and finally, with PBS solution. Antigen unmasking was performed using a TRIS EDTA solution at pH 9 (10 mM Tris-aminomethane (Scharlau, Barcelona, Spain), 1 mM etilendiaminotetraacetic acid (EDTA, Analyticals, Montedison Group, Milan, Italy)) with 0.05% Tween 20 (Fagron, Barcelona, Spain) at 100 °C for 20 min and then washed twice with PBS solution. Then slides were permeabilized with PBS solution with 0.2% Tween 20 (5 min), followed by 30 min of blocking using PBS with bovine serum albumin (BSA 1%) solution. The sections were incubated overnight at 5 °C in a humidity chamber with primary antibodies diluted in blocking solution. The antibodies used in this study were anti-occludin, anti-ZO-1 polyclonal, anti-claudin-2, and anti-claudin-4 polyclonal, all from Thermo Fisher Scientific. The dilutions used were 1:50 for ZO-1 and claudin-4, and 1:100 for occludin and claudin-2. After incubation, the slides were rinsed three times with PBS-0.05% Tween 20 for 10 min. Then, sections were incubated 1 h at 5 °C in a humidity chamber with secondary antibodies. Alexa Fluor 555 donkey anti-rabbit IgG (H+L, Invitrogen, Carlsbad, MA, USA) was diluted 1:1000 in blocking solution. Sections were washed three times with PBS-0.05% Tween 20. Finally, nuclei were stained, sections were incubated 10 min in the humidity chamber with DAPI (1:1000, Invitrogen), then they were rinsed three times with PBS for 10 min and mounted with Fluoromount G™ (Invitrogen). Controls were incubated with secondary antibodies only. 

Images were taken with a fluorescence laser and optical microscope (BX41, Olympus Corporation, Shinjuku, Tokyo, Japan) at 40× magnification and stored in tiff format. The time of exposition was adapted to each staining, but the control images were acquired with the same exposition time. Image analyses and treatments were performed using the ImageJ program. Images that were modified for contrast and brightness to enhance their visualization were processed in the same way as the images corresponding to their respective controls.

### 2.14. Statistical Analysis 

Statistics were performed by the software IBM Statistical Package for the Social Sciences (SPSS, version 22.0, Chicago, IL, USA). The Levene’s test was used to assess the homogeneity of variance and the Shapiro−Wilk test to evaluate the distribution of the results. When there was a normal distribution and equality of variance existed, a conventional one-way ANOVA test was carried out, followed by the Bonferroni post hoc test. On the other hand, results having different variances and/or different distributions were evaluated by the non-parametric Kruskal−Wallis test followed by the Mann−Whitney U post hoc test. Significant differences were established at *p* < 0.05. 

## 3. Results

### 3.1. Body Weight and Other Morphometric Variables

**Figure 1:**
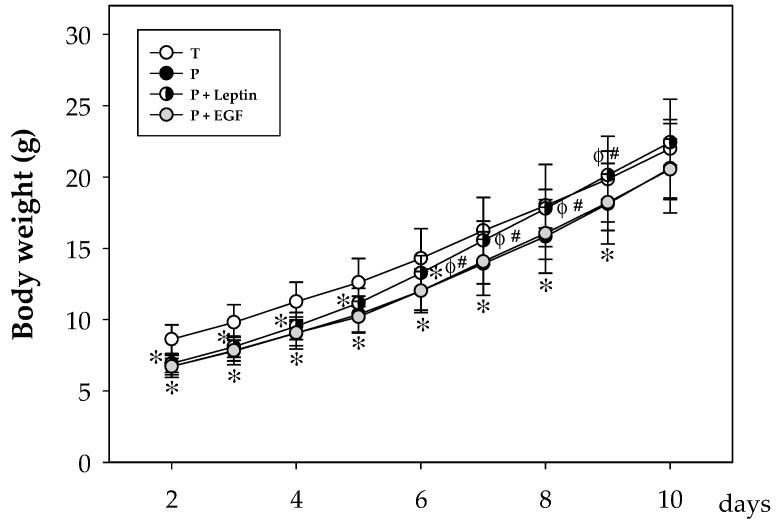
Body weight from the four groups: Term (T), Preterm (P), P+Leptin, and P+epidermal growth factor (P+EGF) the first 10 days of the study. Results are expressed as mean ± SD (*n* = 12–30 pups/group). Statistical differences: * *p* < 0.05 versus T group; ^φ^ *p* < 0.05 versus P group; ^#^ *p* < 0.05 versus P+EGF group.

Body growth assessment in all four groups during the study showed that, as expected, the weight of non-supplemented preterm rats was lower than that of the term pups during the first 9 days (*p* < 0.05), achieving similar values of the T group after this day. Although EGF supplementation to preterm pups was not able to revert this lower weight, animals from the P+Leptin group reached term values at day 7, two days before the P and P+EGF groups did (*p* < 0.05, Figure 1). 

Interestingly, morphometric variables, such as BMI and the Lee index, were also evaluated at day 10 and 17 of the study without observing any changes due to prematurity or supplementation. Although relative weights of the spleen, thymus, and liver were not modified due to prematurity or supplementation, a decrease in relative small and large intestine length was observed in P+Leptin and P+EGF groups at the end of the study (day 17) (*p* < 0.05, Table 1).

### 3.2. Blood Cell Count

On day 10, blood cell count was assessed. No effect due to prematurity or leptin supplementation was observed in the leukocyte count. However, the supplementation with EGF was able to increase the leukocyte cell count due to a higher count of the three populations studied, lymphocytes, monocytes, and granulocytes (*p* < 0.05, versus T, P, and P+Leptin groups, Table 2).

However, preterm rats showed a lower count of erythrocytes compared to the T group (*p* < 0.01, Table 2) and supplementation with both milk bioactive components was not able to revert it (*p* < 0.01 versus T group). Preterm animals had lower hemoglobin (Hb) concentrations and hematocrits (HCT), but higher mean corpuscular hemoglobin (MCH) levels and mean corpuscular volumes (MCVs) (*p* < 0.05 versus T group). Although the count of erythrocytes was not modified in preterm animals supplemented with leptin or EGF, the nutritional interventions were able to revert some of these alterations by inducing a tendency to increase HCTs and to significantly increase Hb concentrations and MCH values (*p* < 0.01 versus P group and *p* < 0.01 versus T and P groups, respectively, Table 2). Moreover, leptin nutritional intervention increased the MCVs (*p* < 0.01 versus P group). Platelet count was not influenced either by prematurity or the nutritional interventions.

### 3.3. Phagocytic Function of Blood Leukocytes

**Figure 2:**
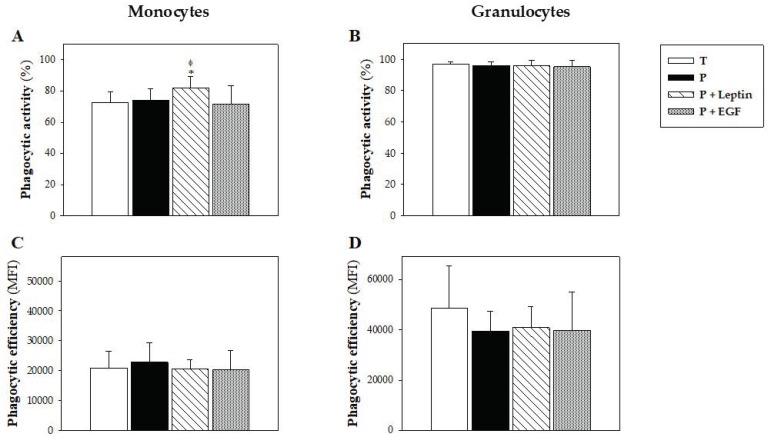
Phagocytic function of blood leukocytes from the four groups: Term (T), Preterm (P), P+Leptin, and P+epidermal growth factor (P+EGF). Phagocytic activity (**A**,**B**) and efficiency (**C**,**D**) at day 10 from monocytes and granulocytes, respectively. Results are expressed as mean ± SD (*n* = 9 pups/group). Statistical differences: * *p* < 0.01 versus T group; ^φ^ *p* < 0.05 versus P group.

The phagocytic activity of blood leukocytes was studied at day 10, focusing on monocytes and granulocytes. Although no changes due to prematurity or supplementation were observed in granulocyte phagocytic activity, animals supplemented with leptin showed higher phagocytic activity in their monocytes compared with the T and P groups (*p* < 0.01, Figure 2A,B). Moreover, the phagocytic efficiency of these leukocytes was not modified by either the prematurity or the supplementation (Figure 2C,D).

### 3.4. Plasma IgA, IgM and IgG Concentrations

**Figure 3:**
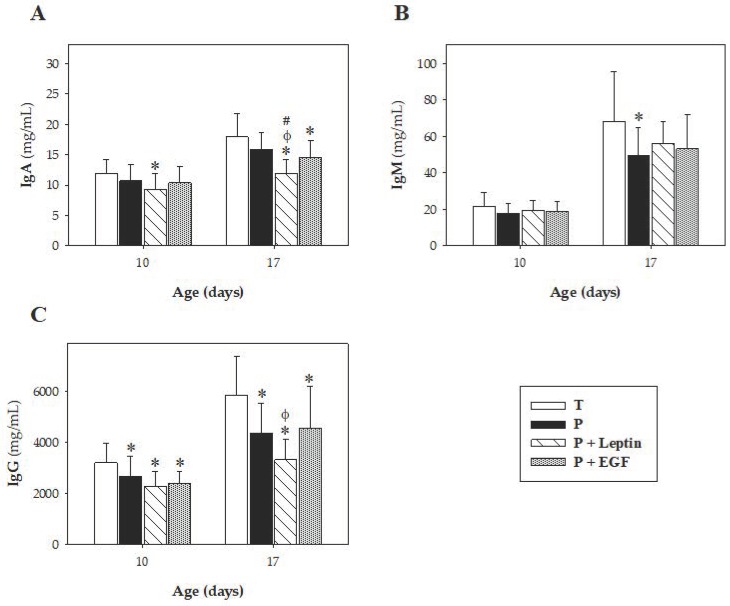
Plasma Ig concentrations at day 10 and 17. Plasma IgA (**A**), IgM (**B**), and IgG (**C**) concentrations from the four groups: Term (T), Preterm (P), P+Leptin, and P+epidermal growth factor (P+EGF), are expressed as mean ± SD (*n* = 8–12 pups/group). Statistical differences: * *p* < 0.05 versus T group; ^φ^ *p* < 0.05 versus P group; # *p* < 0.05 versus P+EGF group.

**Figure 4:**
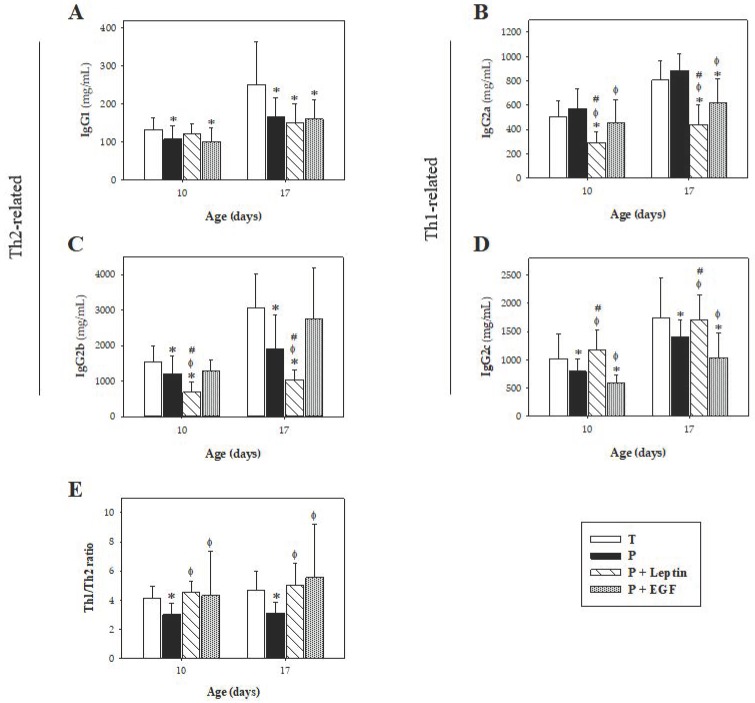
Plasmatic concentration of IgG subclasses at days 10 and 17. IgG1 (**A**), IgG2a (**B**), IgG2b (**C**), IgG2c (**D**), and Th1/Th2 ratios (**E**) from the four groups: Term (T), Preterm (P), P+Leptin, and P+epidermal growth factor (P+EGF) are expressed as mean ± SD (*n* = 8–12 pups/group). Statistical differences: * *p* < 0.05 versus T group; ^φ^ *p* < 0.05 versus P group; # *p* < 0.05 versus P+EGF group.

On days 10 and 17, plasma IgA, IgM, IgG, and IgG isotypes (IgG1, IgG2a, IgG2b, IgG2c, and the Th1/Th2-immune response balance) were quantified and summarized in Figure 3 and Figure 4. In all Ig, a relative, age-associated increase was detected between 10 and 17-day-old rats owing to a normal immune development in all groups (*p* < 0.05, Figure 3).

Although no changes were observed in IgA due to prematurity, leptin and EGF supplementations were able to decrease IgA levels at day 17 (*p* < 0.01 versus T and P group and *p* < 0.05 versus T group, respectively, Figure 3A). In the case of leptin, this significant decrease could already be seen at day 10 (*p* < 0.01 versus T group). Moreover, animals delivered prematurely showed lower concentrations of IgM at day 17 compared to full-term ones (*p* < 0.05, Figure 3B), without modification at day 10. However, supplementation with leptin or EGF was not able to revert this change associated with prematurity.

Regarding IgG and its isotypes, prematurity reduced the concentration of IgG (*p* < 0.01 versus T group, Figure 3C) and three of its isotypes (IgG1, IgG2b, and IgG2c) (*p* < 0.01 versus T group, Figure 4A,C,D) on both days studied, without modifying the IgG2a concentration (Figure 4B). The Th1/Th2 ratio was calculated considering the Th1-related IgG isotypes (IgG2b and IgG2c) and the Th2-related ones (IgG1 and IgG2a) in rats [27,28]. Results showed that prematurity induced lower Th1 antibody response, which was evidenced by a decrease in the Th1/Th2 ratio on both days studied (*p* < 0.01, Figure 4E).

The supplementation with leptin, but not EGF, showed a tendency to increase IgG1 at day 10 (Figure 4A). Nevertheless, at day 17, P+Leptin and P+EGF groups showed similar IgG1 levels to the P group, having lower values compared to the T group (*p* < 0.01). With regard to IgG2a, leptin and EGF supplementations decreased its concentration at day 17 (*p* < 0.01 versus T and P groups) and in the case of leptin, this change could already be detected at day 10 (*p* < 0.01 versus T, P and P+EGF groups, Figure 4B). Moreover, leptin supplementation decreased IgG2b concentration compared with the others studied groups (*p* < 0.01, Figure 4C). Finally, leptin supplementation increased the concentration of IgG2c to levels achieved by term animals (*p* < 0.05 on both days studied versus P and P+EGF groups, Figure 4D), whereas EGF decreased IgG2c levels on both days studied (*p* < 0.01 versus T and P groups, Figure 4D). Overall, the changes produced by leptin and EGF supplementations reverted the Th1/Th2 ratio decrease induced by prematurity on both days studied, inducing a Th1/Th2 ratio similar to that found in the T group (*p* < 0.01 versus P group, Figure 4E). 

### 3.5. Lymphocyte Composition of the Spleen and MLN

**Figure 5:**
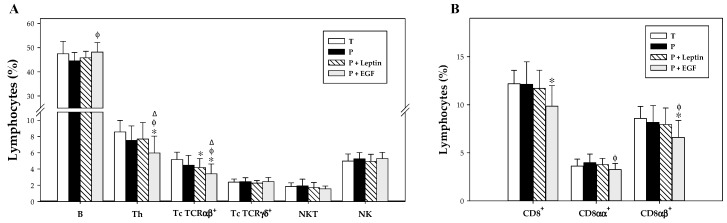
Main lymphocyte subsets (**A**) and CD8^+^ cells and their both forms (CD8αα^+^ and CD8αβ^+^) cell percentages (**B**) in the spleen from the four groups: Term (T), Preterm (P), P+Leptin, and P+epidermal growth factor (P+EGF) at day 17. The results are expressed as mean ± SD (*n* = 8–12 pups/group). Statistical differences: * *p* < 0.05 versus T group; ^φ^ *p* < 0.05 versus P group; ^Δ^ *p* < 0.05 versus P+Leptin group.

**Figure 6:**
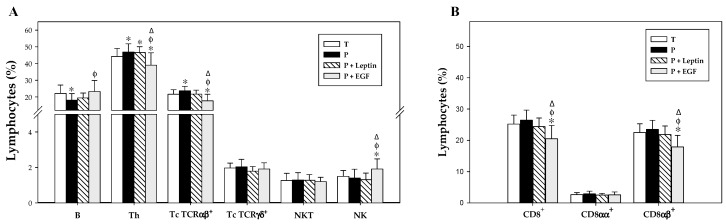
Main lymphocyte subsets (**A**) and CD8^+^cells and their both forms (CD8αα^+^ and CD8αβ^+^) cell percentages (**B**) in the mesenteric lymph nodes (MLNs) from the four groups: Term (T), Preterm (P), P+Leptin, and P+epidermal growth factor (P+EGF) at day 17. The results are expressed as mean ± SD (*n* = 8–12 pups/group). Statistical differences: * *p* < 0.05 versus T group; ^φ^ *p* < 0.05 versus P group; ^Δ^ *p* < 0.05 versus P+Leptin group.

At day 17, the lymphocyte pattern was characterized in the spleen and MLN and summarized in Figure 5 and Figure 6. The main population present in the spleen was B cells and the percentages of the rest of the populations were lower (Figure 5). On the other hand, these percentages changed in the MLN, where the main population was Th, followed by B cells and Tc TCRαβ^+^; and in lower proportions, Tc TCRγδ^+^, NK, and NKT cells (Figure 6). 

In the spleen, although no changes in phenotypical composition appeared due to prematurity, a tendency to decrease the proportion of B, Th, and Tc TCRαβ^+^ cells was observed, but without reaching statistical significance (Figure 5A). Leptin supplementation for 17 days was not able to revert these effects induced by prematurity, and in addition, it decreased the percentage of Tc TCRαβ^+^ cells even more (*p* < 0.05). However, animals supplemented with EGF increased the splenic B cell proportion to similar levels of those of the T group (*p* < 0.01 versus P group, Figure 5A). Moreover, a decrease in Th and Tc TCRαβ^+^ was observed due to EGF supplementation (*p* < 0.05 versus T, P, and P+Leptin groups, Figure 5A). In regard to the CD8 co-receptor, no changes were observed on splenocytes on account of prematurity or leptin supplementation. Nevertheless, EGF supplementation decreased the CD8^+^ cell percentage (*p* < 0.01, Figure 5B). This change was due to a reduction in the proportion of both CD8αα^+^ and CD8αβ^+^ cells (*p* < 0.05, Figure 5B).

Regarding lymphocytes from MLN, despite there being no significant changes observed in the Tc TCRγδ^+^, NKT, NK, and CD8^+^ subsets of premature rats with respect to term ones, a decrease in B cell percentages and an increase in Th and Tc TCRαβ^+^ proportions were observed in non-supplemented preterm rats (*p* < 0.05 versus T group, Figure 6). Leptin supplementation for 17 days did not produce any significant effect on the lymphocyte composition of the MLN. However, EGF increased B cell percentages (*p* < 0.05 versus P group, Figure 6A) and decreased Th and Tc TCRαβ^+^ cell proportions (*p* < 0.05 versus T, P, and P+Leptin group), with the same tendency observed in splenocytes. Moreover, an increase in the NK subset was observed in rats supplemented with EGF (*p* < 0.05 versus T, P, and P+Leptin groups, Figure 6A). Furthermore, the CD8^+^ lymphocyte proportion in MLN was not affected by prematurity or leptin supplementation, but EGF supplementation for 17 days was able to decrease its percentage. This decrease was caused by a reduction in CD8αβ^+^ cell percentage (*p* < 0.01 versus T, P, and P+Leptin groups).

### 3.6. Intestinal Barrier Function

**Figure 7:**
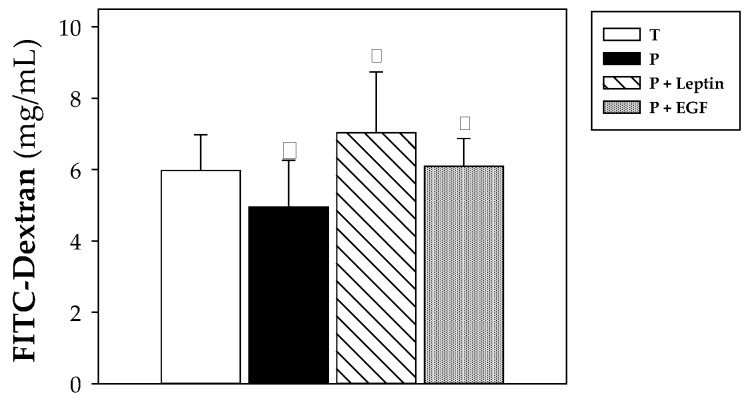
Intestinal permeability to 4 kDa-FITC-dextran from the four groups: Term (T), Preterm (P), P+Leptin, and P+epidermal growth factor (P+EGF) at day 10. Results are expressed as mean ± SD (*n* = 9 pups/group). Statistical differences: * *p* < 0.05 versus T group; ^φ^ *p* < 0.05 versus P group.

To study the intestinal permeability, an in vivo assay was performed evaluating the paracellular pass of the 4 kDa-dextran labeled with FITC at day 10 (Figure 7). Premature animals had lower intestinal permeability, showing a lower concentration of FITC dextran in their plasma compared to that found in animals from T group (*p* < 0.05, Figure 7). Leptin and EGF supplementation for 10 days was able to revert this effect, reaching FITC-dextran levels similar to those observed in term rats (*p* < 0.05 in P+Leptin and P+EGF groups versus P group, Figure 7).

### 3.7. Intestinal Histomorphometric Study

**Figure 8:**
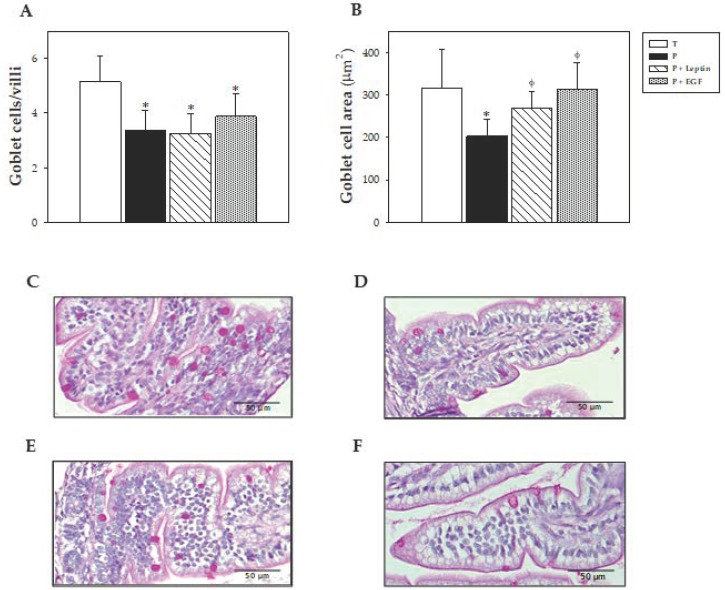
Number of goblet cells/villi (**A**), goblet cells area (**B**), and representative images of histological sections of the jejunum with periodic acid−Schiff (PAS) staining from the four groups: Term (**C**), Preterm (**D**), P+Leptin (**E**), and P+epidermal growth factor (P+EGF) (**F**) at day 10 of the suckling period. Results of Figure 8A,B are expressed as mean ± SD (*n* = 6 pups/group). Statistical differences: * *p* < 0.05 versus T group; ^φ^ *p* < 0.05 versus P group. Goblet cells with densely stained granules can be observed along the length of the villi (Figure 8C–F). Scale bar = 50 μm for 400 ×.

At day 10, the morphology of the distal jejunum was also evaluated, focusing on intestinal villi and goblet cell characteristics. The variables of the villi studied—widths, heights, and perimeters—are summarized in Table 3. No histological differences due to prematurity or supplementations were observed in the villi’s variables. In regard to the goblet cells, animals born in preterm conditions showed a lower number of the cells in the villi, which, in addition, were smaller (*p* < 0.05 versus T group, Figure 8). Although the P+Leptin and P+EGF group also exhibited a lower number of goblet cells (*p* < 0.05 versus T group), they had a similar area with respect to the T group, reversing the prematurity effect (*p* < 0.05 versus P group, Figure 8).

### 3.8. Intestinal Gene Expression

**Figure 9:**
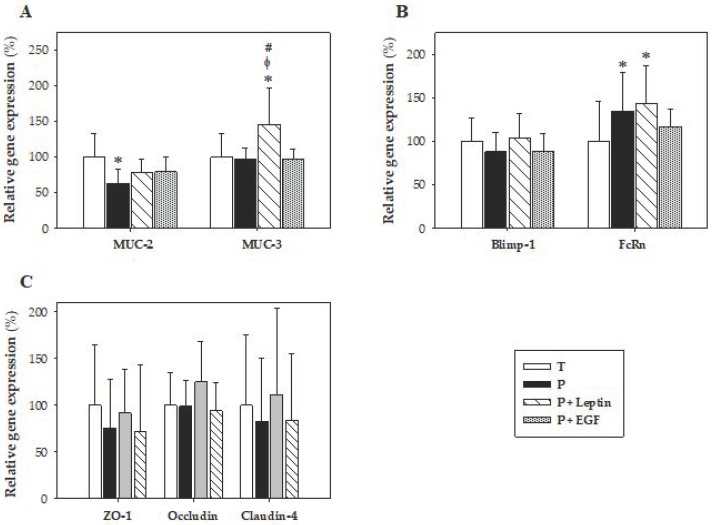
Gene expression in small intestine samples from the four groups: Term (T), Preterm (P), P+Leptin, and P+epidermal growth factor (P+EGF) at day 10 of the suckling period. *MUC-2* and *MUC-3* (**A**), *Blimp-1* and *FcRn* (**B**), *ZO-1*, *occludin*, and *claudin-4* (**C**). Results are expressed as mean ± SD (*n* = 9 pups/group). Statistical differences: * *p* < 0.05 versus T group; ^φ^ *p* < 0.05 versus P group; # *p* < 0.05 versus P+EGF group.

The gene expression of proteins involved in mucus production, such as MUC-2 and MUC-3; molecules used as biomarkers of intestinal maturation, such as Blimp-1 and FcRn; and proteins from tight junctions, such as ZO-1, occluding, and claudin-4, were evaluated at day 10 (Figure 9). In regard to the expression of mucins, premature animals had lower *MUC-2* gene expression without changes in the *MUC-3* gene (*p* < 0.01 versus T group, Figure 9A). Leptin supplementation increased both mucins’ gene expression; however, this increase was only significant for *MUC-3* (*p* < 0.01 versus T, P, and P+EGF groups, Figure 9A). No changes were observed after the EGF supplementation. 

With reference to the gene expression of the intestinal maturation biomarkers Blimp-1 and FcRn, an increase in *FcRn* was observed in preterm rats, and leptin was not able to revert this effect, showing values similar to the P group (*p* < 0.01 versus T group, Figure 9B). No significant differences were detected between groups on *Blimp-1* gene expression. Moreover, the gene expression of tight junction proteins was also analyzed, but they were not modified by either the prematurity or the supplementation (Figure 9C). EGF did not induce any effect at the expression levels of the genes studied.

### 3.9. Intestinal Immunofluorescent Study

**Figure 10:**
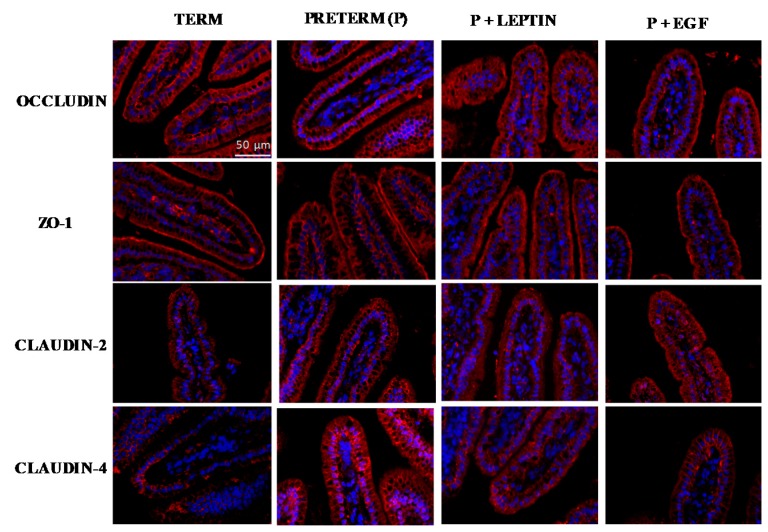
Immunofluorescent staining of small intestine tissue for occludin, ZO-1, claudin-2, and claudin-4. Representative images from Term (T), Preterm (P), P+Leptin, and P+epidermal growth factor (P+EGF) intestines at day 10. Similar results were obtained in five animals in each group. Nuclei were stained with DAPI (blue). Localization of occludin, ZO-1, claudin-2, and claudin-4 (red) were observed by fluorescence microscopy at 400× magnification. Scale bar: 50 µm.

Immunofluorescent staining was performed in order to observe the changes in the distribution of occludin, ZO-1 and claudin-2 and -4 due to prematurity or nutritional interventions. The staining pattern of occludin was similar among studied groups and localized in the apical membrane (Figure 10). A delocalization of ZO-1 in the P group was observed compared to the T group. Nevertheless, P+EGF reached a similar staining pattern to the T group. In addition, claudin-2 and -4 were also studied. Both T and P groups showed a similar staining pattern to claudin-2 but P+EGF showed an increase in fluorescence levels with respect to the T group. Moreover, claudin-4 showed an increase in fluorescence stain in premature rats with respect to term rats (Figure 10). In regard to supplementations, P+Leptin and P+EGF showed a staining pattern similar to that of the T group.

## 4. Discussion

We have previously reported that in full-term rats the supplementation with leptin and EGF during the whole suckling period results in an enhancement of both the systemic and intestinal immune system [21,22,23]. However, little is known about the activity of these compounds in the naïve immune system of the preterm infant. These results prompted us to study whether those effects could also be observed in the rats born in preterm conditions. For that, a preterm rat model, recently set up in our laboratory, was used [24]. To achieve our aims, preterm rats born by C-section were supplemented with leptin and EGF during the first 17 days of life. The data presented here demonstrate that the supplementation with leptin was able to increase the hemoglobin concentration, to enhance phagocytic activity of monocytes, to modify plasma Ig concentration to a Th1 pattern, to revert the premature effect on intestinal permeability, to increase goblet cells’ area, and to increase *MUC-3* gene expression. In addition, EGF supplementation increased both leukocyte and erythrocyte counts, decreased Th, T TCRαβ^+^ and CD8^+^ cell percentages, and increased the B cell proportion in MLNs and the spleen. Moreover, both milk components reversed prematurity’s effects on intestinal permeability and goblet cell size.

With regards to body growth, the body weight of premature pups was lower than full-term ones only during the first 9 days, with no differences detected between them after that day. These results were similar to those observed in the preterm rat model, for which lower weight was observed during the whole study (10 days) [24], confirming in our model, the well-known observation that the length of gestation period has an effect on body weight. Although EGF-supplemented rats showed the same pattern as the P group, interestingly, leptin-supplemented pups were able to achieve the weight of the T group two days before the P group. Although a weight loss was expected, given the satiating role of leptin described in adults [29,30], no changes in body weight were observed in previous studies performed with rats supplemented with leptin during the whole suckling period [21,31]. However, it has to be taken into account that those rats were born at term, while in our study they were born prematurely. Moreover, no differences were found between BMIs, Lee indices, or organ relative weights. The punctual decrease observed in the relative length of small and large intestine due to leptin and EGF supplementations was not in line with that found in other studies performed in term rats [21,23,32]. Moreover, there is no information regarding the impact of leptin or EGF on the intestinal growth in preterm conditions. Therefore, further studies are needed to better understand the role of these components in the context of prematurity.

Focusing on the blood cell count, the preterm rats showed lower counts of erythrocytes, and lower Hb concentrations and HCTs compared to term ones. In addition, they showed higher MCH levels and MCVs, suggesting the presence of a macrocytic hyperchromic anemia. This result was in line with the macrocytic anemia observed in humans and in our previous study in preterm rats [24,33]. Premature infants may have anemia due to multiple factors, such as rapid body growth, low plasma levels of erythropoietin, or inadequate nutrient intake, among others [34,35]. Although the supplementation with leptin and EGF was not able to revert the erythrocyte count, the MCV or MCH, it was able to increase the mean Hb concentration compared to the P group. Little is known about the effect of leptin or EGF in erythropoiesis and even less in prematurity, but it might be a mechanism to compensate the reduced number of erythrocytes. However, the effect of EGF raising the leukocyte count could be explained by its role in hematopoiesis, increasing cellular proliferation and decreasing the apoptosis of hematopoietic stem cells in mice [36,37].

Newborns have a very immature immune system, due to a reduced adaptive immune response because of their lack of contact with pathogens until birth. Thus, the infant is mainly protected by an immature innate immunity. In our study, although no differences were observed in both phagocytic activity and the efficiency of both monocytes and granulocytes because of the prematurity and EGF supplementation. Rats supplemented with leptin showed a higher phagocytic activity of their monocytes. This result is in accordance with the role of leptin in enhancing the phagocytic activity of monocytes/macrophages in adult rodents [38,39]. This increase might be beneficial to the preterm newborn, since it implies an early maturation of the innate immune response.

In preterm animals, the concentrations of IgM and IgG were lower than T group. In line with our results, it has been described that human preterm infants have lower serum IgM and IgG concentrations, which, in addition, correlates positively with the gestational age and the weight of the baby at the time of birth [40]. The lower IgG concentration was not reverted by these breast milk components. Moreover, the supplementation with leptin and EGF reduced the plasma IgA concentration on both days studied and at day 17, respectively. It has been previously described that B cells have leptin receptors on their surfaces, suggesting a direct effect of leptin on B cell function [39]. However, little is known about the effect of leptin or EGF on IgA secretion. Nevertheless, we have previously reported the ability of leptin to decrease IgA in the intestinal compartment at the end of the suckling period in full-term rats [21]. Nevertheless, it must be taken into account that the main concentration of IgA detected in the intestine compartment comes from the mother’s milk and not from the newborn. Further studies must be directed to ascertain the mechanisms involved in this decrease due to leptin supplementation. 

In regard to IgG isotypes, this model of prematurity induced a reduction in both Th1-related IgG (IgG2b and IgG2c) and Th2-related IgG (IgG1), reducing the Th1/Th2 ratio compared with rats that were born at term. This result is in accordance with the bias towards a Th2 response described for preterm humans [41], making preterm infants more vulnerable to infections. Leptin is suggested to enhance the Th1 response and to inhibit the Th2 one [19,42]. In our study, with the exception of IgG2b, a decrease in both IgG1 and IgG2a, and an increase in IgG2c, was observed. Thus, these changes counteract the Th1/Th2 imbalance present in preterm animals. On the other hand, little is known about the effect of EGF on IgG isotypes and we reported here, the influence of this growth factor on IgG1, IgG2a, IgG2b, and IgG2c. Preterm rats supplemented with EGF showed a decrease in IgG1, IgG2a, and IgG2c, increasing the Th1/Th2 ratio. These effects suggest that leptin and EGF might enhance the Th1 response, improving the Th1/Th2 balance, representative of later stages. Thus, both supplementations seem to be involved in promoting the adequate initial immune homeostasis induced by breast milk that encourages a shift from an intrauterine Th2 predominant response to a Th1/Th2 balanced response [43,44].

In the present study, we have described for the first time, the effect of prematurity on the rat lymphocyte composition in the spleen and MLN, and also the impact of a supplementation with leptin or EGF on its composition. It has been previously described that preterm infants have a lower absolute number of B, Th, Tc, and NK cells in cord blood compared to term ones [45,46]. On the contrary, Correa-Rocha et al. reported that premature infants had a higher percentage of Th and Tc and a lower percentage of NK and B cells in cord blood [45]. Similar results in lymphocyte composition were observed in our study, where the prematurity condition lowered the proportion of B cells and raised those of Th and Tc TCRαβ^+^ cells in MLNs at day 17. Nevertheless, no changes were observed in the lymphocyte pattern in the spleen. 

Regarding the influence of milk bioactive components on the lymphocyte composition at day 17, leptin supplementation did not reverse any of the effects induced by prematurity in either studied compartment. Nevertheless, rats supplemented with EGF showed higher levels of B cells and lower percentages of Th and Tc TCRαβ^+^ in both studied lymphoid organs, modifying premature changes observed in MLNs. Little is known about the effect of this growth factor on the spleen lymphocyte composition. So, these results are a first approach to elucidate the role of this growth factor modulating lymphocyte pattern in prematurity. Furthermore, in MLNs, EGF was also able to increase NK cell percentage at day 17. Little is known about the effect of EGF on NK cells, but the enhancement of this population in early life could be beneficial to the preterm infants who have a higher rate of infections due to their immature immune systems [41]. Regarding the cells bearing CD8 molecules, EGF was able to decrease that cell proportion in premature rats, in both compartments, but acting in different ways. While the diminution of the CD8^+^ percentage was due to a lower proportion of CD8αβ^+^ in the MLNs, the decrease in the spleen was caused by both forms of CD8 co-receptor (CD8αα and CD8αβ). However, the influence of EGF in the CD8^+^ cell population has scarcely been explored. 

Those changes in phenotypical lymphocyte proportions in both the spleen and MLNs due to leptin and EGF supplementation would also lead to a modification in their patterns of secretion of pro-inflammatory and anti-inflammatory cytokines. Following on, in previous studies in term rats supplemented with these bioactive compounds, we observed changes in the cytokines released by cultured lymphocytes from MLNs and spleen [21,22,23]. Particularly, leptin was able to promote an anti-inflammatory pattern of cytokines [21,22], whereas EGF induced the release of pro-inflammatory cytokines [23]. 

At day 10 of the suckling period, the intestinal barrier function was studied by means of the evaluation of the permeability to 4-kDa-dextran. Previous studies carried out in our laboratory reported a decrease in intestinal permeability due to prematurity [24]. This unexpected output was also observed in the present study, thus confirming this result. The supplementation with leptin or EGF for 10 days was able to revert this effect and increase the intestinal permeability to values observed in full-term rats. Supporting this result, an increase in the intestinal paracellular permeability after an intraperitoneal injection of leptin was also reported in rats [47]. Nevertheless, that suggests the outcome of the review that EGF plays an important role in regulating intestinal permeability and epithelial barrier integrity [48]. In this sense, Clark et al. described that EGF treatment decreased intestinal paracellular permeability, reducing the concentration of lactulose in blood compared to the control group [49].

In the intestinal histomorphometric study, no changes were observed in the variables studied in the villi between groups. In respect to cells, the three preterm groups showed a lower number of goblet cells, and in the case of the P group, they were of a smaller size than those from the T group. However, leptin and EGF supplementations were able to increase goblet cell size. In this regard, it has been reported that a smaller size and density of the goblet cells could be related to immaturity, producing less mucus at the intestinal barrier level [49]. Thus, leptin and EGF could promote the maturation of these cells, improving mucus production. Moreover, it has been described that a supplementation with EGF increased goblet cell density and mucin production [49].

Goblet cells are specialized epithelial cells that play an important role in the intestine by synthesizing and secreting mucus, including MUC-2, which is responsible for the gel-forming mucins [50]. Although no changes were observed in *MUC-3* gene expression, premature rats showed lower *MUC-2* levels than full-term ones. So, the decrease in *MUC-2* gene expression could be related to the lower number and size of goblet cells observed in the histomorphometric study. Moreover, the tendency to increase *MUC-2* gene expression by leptin supplementation might be related with its capacity to increase goblet cell size. Moreover, although prematurity did not modify *MUC-3* gene expression, nutritional intervention with leptin was able to increase its expression. This effect was previously observed in rats after leptin supplementation during suckling, after a perfusion of leptin in the rat’s colon or after adding leptin in an *in vitro* study [2]. EGF was previously reported to increase *MUC-2* gene expression [49]. However, this result was not observed in our study. Therefore, more studies are needed to elucidate the role of this growth factor on the expression of mucins.

The neonatal Fc receptor (FcRn) mediates the transfer of IgG from the placenta to the fetus, and from suckled maternal milk to the neonatal circulation [51]. The expression of this gene is a good biomarker of the process of intestinal maturation in lactating rats because it is highly expressed in early life and decreases at weaning [52]. Thus, a higher expression of this gene in preterm rats could mean a higher intestinal immaturity compared to the term ones. However, leptin or EGF supplementation was not able to revert this change. Neither the prematurity nor the supplementations modified *Blimp-1* gene expression, suggesting that neither of them have a role in the expression of this gene, which is also associated with maturation, in premature conditions.

The formation and localization of tight junctions is important for the maintenance of intestinal permeability, and for the epithelial barrier function [49]. For this reason, gene expressions of *ZO-1*, *occludin* and *claudin-4* were studied. However, no differences were found in their gene expressions due to prematurity or supplementations. Nevertheless, other approaches show the involvement of those proteins in related functions. Subsequently, in an *in vitro* study, the addition of 100 ng/mL of leptin to Caco-2 BBe cell culture decreased *occludin* gene expression [53]. Regarding EGF, Xu et al. showed that its oral administration to early weaned piglets promoted *ZO-1*’s and *occludin*’s gene expressions [54]. In addition, another study demonstrated that a supplementation with EGF was able to increase *occludin*’s gene expression in rats with NEC [49].

To gain more in-depth knowledge about tight junction proteins, an immunofluorescent staining of the small intestine was performed. ZO-1 has an important role in tight-junction assembly, while claudin-4 is a key molecule for barrier-forming [55]. Takehara et al. reported that higher expression of claudin-4 is related to an increased paracellular permeability [56]. Supporting that result, in our study non-supplemented preterm rats that had higher permeability showed an increase in claudin-4 fluorescent intensity compared to term rats. Moreover, rats supplemented with leptin or EGF showed lower permeability, and their claudin-4 fluorescence was similar to animals born at term. Furthermore, the supplementation with EGF showed an increased fluorescence of claudin-2. Little is known about the effect of EGF on this tight-junction protein; however, in NEC neonatal mice models, animals with NEC had increased claudin-2 expression and intestinal permeability [57]. Therefore, the increase in claudin-2 expression could be related with the higher permeability found in the EGF-supplemented rats. Further studies are needed to better understand the role of these components on tight-junction proteins.

## 5. Conclusions

Overall, the results obtained in this study show that prematurity produces, among other changes, a delay in the maturation of the neonatal immune system. However, the daily supplementation with leptin or EGF during the suckling period is able to partially counteract some of these changes: specifically, some aspects of both the systemic and intestinal immune system of preterm rats, reinforcing the role of both bioactive components of human milk in terms of modulating the intestinal barrier’s function and the immune response in early life.

## Figures and Tables

**Table 1:** Morphometric variables and relative organ weights or lengths from Term (T), Preterm (P), P+Leptin, and P+epidermal growth factor (P+EGF) groups at days 10 and 17.

	**Day 10**			
	**T**	**P**	**P+Leptin**	**P+EGF**
**BMI** (g/cm^2^)	0.33 ± 0.02	0.34 ± 0.02	0.34 ± 0.02	0.34 ± 0.02
**Lee Index** ((^3^√g/cm) × 1000)	337.51 ± 7.59	341.57 ± 5.00	337.19 ± 6.45	341.13 ± 8.85
**Spleen weight** (%)	0.57 ± 0.10	0.59 ± 0.09	0.62 ± 0.07	0.53 ± 0.21
**Thymus weight** (%)	0.34 ± 0.07	0.33 ± 0.06	0.32 ± 0. 13	0.39 ± 0.04
**Liver weight** (%)	3.21 ± 0.29	3.45 ± 0.39	3.41 ± 0.14	3.25 ± 0.41
**Small intestine length** (%)	163.90 ± 20.83	171.31 ± 20.45	158.78 ± 17.00	156.19 ± 19.43
**Large intestine length** (%)	19.07 ± 2.57	18.75 ± 2.64	17.11 ± 1.63	19.25 ± 2.44
	**Day 17**			
**BMI** (g/cm^2^)	0.38 ± 0.02	0.39 ± 0.04	0.39 ± 0.01	0.41 ± 0.03
**Lee Index** ((^3^√g/cm) × 1000)	327.61 ± 5.67	329.46 ± 13.07	329.72 ± 6.64	336.63 ± 9.34
**Spleen weight** (%)	0.49 ± 0.05	0.54 ± 0.06	0.55 ± 0.06	0.51 ± 0.16
**Thymus weight** (%)	0.41 ± 0.08	0.42 ± 0.07	0.43 ± 0.09	0.44 ± 0.06
**Liver weight** (%)	3.82 ± 0.21	3.87 ± 0.34	3.70 ± 0.30	3.82 ± 0.31
**Small intestine length** (%)	93.08 ± 11.15	94.67 ± 7.23	84.36 ± 10.30 * ^φ^	89.06 ± 10.56 ^φ^
**Large intestine length** (%)	14.25 ± 2.12	13.75 ± 2.86	12.50 ± 1.92 *	12.38 ± 2.46 *

Relative organ weight or length was calculated as weight or length of the organ divided by the body weight × 100. Data are expressed as mean ± SD (*n* = 9–12 pups/group). Statistical differences: * *p* < 0.05 versus T group; ^φ^ *p* < 0.05 versus P group. BMI: body mass index.

**Table 2:** Blood cell count from the four groups: Term (T), Preterm (P), P+Leptin, and P+epidermal growth factor (P+EGF) at day 10 of the suckling period.

	**Day 10**			
	**T**	**P**	**P+Leptin**	**P+EGF**
**Leukocytes** (×10^9^/L)	2.48 ± 0.58	2.45 ± 0.68	2.52 ± 0.45	3.57 ± 1.31 *^φ Δ^
**Lymphocytes** (×10^9^/L)	1.71 ± 0.49	1.65 ± 0.50	1.74 ± 0.48	2.41 ± 1.02 *^φ Δ^
**Monocytes** (×10^9^/L)	0.23 ± 0.07	0.21 ± 0.07	0.22 ± 0.05	0.31 ± 0.10 *^φ Δ^
**Granulocytes** (×10^9^/L)	0.54 ± 0.20	0.59 ± 0.31	0.55 ± 0.20	0.85 ± 0.39 *^φ Δ^
**Erythrocytes** (×10^12^/L)	3.41 ± 0.25	3.03 ± 0.21 *	3.12 ± 0.22 *	3.10 ± 0.33 *
**Hb** (g/L)	82.90 ± 5.75	78.37 ± 5.13 *	85.52 ± 5.27 ^φ^	82.94 ± 6.48 ^φ^
**HCT** (%)	28.95 ± 2.02	27.09 ± 2.45 *	28.53 ± 2.54	27.93 ± 2.08
**MCV** (fL)	86.22 ± 3.40	90.74 ± 3.95 *	92.01 ± 4.08 ^φ^	90.04 ± 4.08 *
**MCH** (pg)	24.64 ± 1.04	25.83 ± 1.37 *	27.48 ± 2.00 *^φ^	26.71 ± 1.94 *^φ^
**Platelets** (× 10^12^/L)	467.45 ± 81.99	502.94 ± 89.08	514.44 ± 63.64	455.25 ± 96.91

The results are expressed as mean ± SD (*n* = 18 pups/group). Statistical differences: * *p* < 0.05 versus T group; ^φ^ *p* < 0.05 versus P group; ^Δ^ *p* < 0.05 versus P+Leptin group. Hb: hemoglobin; HCT: hematocrit, MCV: mean corpuscular volume; MCH: mean corpuscular hemoglobin.

**Table 3:** Histomorphometric variables of the small intestine: villi widths, heights, and perimeters from the four groups: Term (T), Preterm (P), P+Leptin, and P+epidermal growth factor (P+EGF) at day 10 of suckling period.

	T	P	P+Leptin	P+EGF
Villi width (μm)	165.91 ± 29.73	157.70 ± 29.17	166.96 ± 12.67	157.19 ± 32.49
Villi height (μm)	571.40 ± 104.95	547.55 ± 41.60	502.00 ± 49.46	487.76 ± 75.86
Villi perimeter (μm)	1223.07 ± 181.95	1213.25 ± 115.10	1176.00 ± 148.57	1102.18 ± 184.14

The results are expressed as a mean ± SD (*n* = 6 pups/group).

## References

[1] Boquien C.Y. (2018). Human milk: An ideal food for nutrition of preterm newborn. Front. Pediatrics.

[2] El Homsi M., Ducroc R., Claustre J., Jourdan G., Gertler A., Estienne M., Bado A., Scoazec J.Y., Plaisancié P. (2007). Leptin modulates the expression of secreted and membrane-associated mucins in colonic epithelial cells by targeting PKC, PI3K, and MAPK pathways. Am. J. Physiol. Liver Physiol..

[3] Neu J. (2007). Gastrointestinal development and meeting the nutritional needs of premature infants. Am. J. Clin. Nutr..

[4] Gartner L.M., Morton J., Lawrence R.A., Naylor A.J., O’Hare D., Schanler R.J., Eidelman A.I., American Academy of Pediatrics Section on Breastfeeding (2005). Breastfeeding and the use of human milk. Pediatrics.

[5] Underwood M.A. (2013). Human milk for the premature infant. Pediatrics Clin. North Am..

[6] Garcia C., Duan R.D., Brévaut-Malaty V., Gire C., Millet V., Simeoni U., Bernard M., Armand M. (2013). Bioactive compounds in human milk and intestinal health and maturity in preterm newborn: An overview. Cell. Mol. Biol..

[7] Ehrenkranz R.A. (2010). Early nutritional support and outcomes in ELBW infants. Early Hum. Dev..

[8] Rochow N., Landau-Crangle E., Fusch C. (2015). Challenges in breast milk fortification for preterm infants. Curr. Opin. Clin. Nutr. Metab. Care.

[9] Sisk P.M., Lovelady C.A., Dillard R.G., Gruber K.J., O’Shea T.M. (2007). Early human milk feeding is associated with a lower risk of necrotizing enterocolitis in very low birth weight infants. J. Perinatol..

[10] Meinzen-Derr J., Poindexter B., Wrage L., Morrow A.L., Stoll B., Donovan E.F. (2009). Role of human milk in extremely low birth weight infants’ risk of necrotizing enterocolitis or death. J. Perinatol..

[11] Cacho N.T., Parker L.A., Neu J. (2017). Necrotizing enterocolitis and human milk feeding: A systematic review. Clin. Perinatol..

[12] Patel A.L., Kim J.H. (2018). Human milk and necrotizing enterocolitis. Semin. Pediatrics Surg..

[13] Isaacs E.B., Fischl B.R., Quinn B.T., Chong W.K., Gadian D.G., Lucas A. (2010). Impact of breast milk on intelligence quotient, brain size, and white matter development. Pediatrics Res..

[14] van Odijk J., Kull I., Borres M.P., Brandtzaeg P., Edberg U., Hanson L.A., Høst A., Kuitunen M., Olsen S.F., Skerfving S. (2003). Breatfeedig and allergic disease: A multidisciplinary review of the literature (1966–2001) on the mode of early feeding in infancy and its impact on later atopic manifestations. Allergy.

[15] Vohr B.R., Poindexter B.B., Dusick A.M., McKinley L.T., Wright L.L., Langer J.C., Poole W.K., NICHD Neonatal Research Network (2006). Beneficial effects of breast milk in the neonatal intensive care unit on the developmental outcome of extremely low birth weight infants at 18 months of age. Pediatrics.

[16] Section on Breastfeeding (2012). Breastfeeding and the use of human milk. Pediatrics.

[17] Gidrewicz D.A., Fenton T.R. (2014). A systematic review and meta-analysis of the nutrient content of preterm and term breast milk. BMC Pediatrics.

[18] Lewis E.D., Richard C., Larsen B.M., Field C.J. (2017). The importance of human milk for immunity in preterm infants. Clin. Perinatol..

[19] Carbone F., La Rocca C., Matarese G. (2012). Immunological functions of leptin and adiponectin. Biochimie.

[20] Field C.J. (2005). The immunological components of human milk and their effect on immune development in infants. J. Nutr..

[21] Grases-Pintó B., Abril-Gil M., Rodríguez-Lagunas M.J., Castell M., Pérez-Cano F.J., Franch À. (2018). Leptin and adiponectin supplementation modifies mesenteric lymph node lymphocyte composition and functionality in suckling rats. Br. J. Nutr..

[22] Grases-Pintó B., Abril-Gil M., Castell M., Pérez-Cano F.J., Franch À. (2019). Enhancement of immune maturation in suckling rats by leptin and adiponectin supplementation. Sci. Rep..

[23] Torres-Castro P., Abril-Gil M., Rodríguez-Lagunas M.J., Castell M., Pérez-Cano F.J., Franch À. (2018). TGF-β2, EGF, and FGF21 growth factors present in breast milk promote mesenteric lymph node lymphocytes maturation in suckling rats. Nutrients.

[24] Grases-Pintó B., Torres-Castro P., Abril-Gil M., Castell M., Rodríguez-Lagunas M.J., Pérez-Cano F.J., Franch À. (2019). A preterm rat model for immunonutritional studies. Nutrients.

[25] Azagra-Boronat I., Massot-Cladera M., Knipping K., Van’t Land B., Stahl B., Garssen J., Rodríguez-Lagunas M.J., Franch À., Castell M., Pérez-Cano F.J. (2018). Supplementation with 2′-FL and scGOS/lcFOS ameliorates rotavirus-induced diarrhea in suckling rats. Front. Cell. Infect. Microbiol..

[26] Camps-Bossacoma M., Abril-Gil M., Franch À., Pérez-Cano F.J., Castell M. (2014). Induction of an oral sensitization model in rats. Clin. Immunol. Endocr. Metab. Drugs.

[27] Gracie J.A., Bradley J.A. (1996). Interleukin-12 induces interferon-gamma-dependent switching of IgG alloantibody subclass. Eur. J. Immunol..

[28] Saoudi A., Bernard I., Hoedemaekers A., Cautain B., Martinez K., Druet P., De Baets M., Guéry J.C. (1999). Experimental autoimmune myasthenia gravis may occur in the context of a polarized Th1- or Th2-type immune response in rats. J. Immunol..

[29] Ahima R.S., Prabakaran D., Mantzoros C., Qu D., Lowell B., Maratos-Flier E., Flier J.S. (1996). Role of leptin in the neuroendocrine response to fasting. Nature.

[30] Friedman J.M., Halaas J.L. (1998). Leptin and the regulation of body weight in mammals. Nature.

[31] Picó C., Oliver P., Sánchez J., Miralles O., Caimari A., Priego T., Palou A. (2007). The intake of physiological doses of leptin during lactation in rats prevents obesity in later life. Int. J. Obes. (Lond.).

[32] Hormi K., Lehy T. (1996). Transforming growth factor-α in vivo stimulates epithelial cell proliferation in digestive tissues of suckling rats. Gut.

[33] Hoffbrand A.V. (1970). Folate deficiency in premature infants. Arch. Dis. Child..

[34] Widness J.A. (2010). Pathophysiology of anemia during the neonatal period, including anemia of prematurity. Neoreviews.

[35] Strauss R.G. (2010). Anaemia of prematurity: Pathophysiology and treatment. Blood Rev..

[36] Piryani S.O., Kam A.Y.F., Kliassov E.G., Chen B.J., Spector N.L., Chute J.P., Hsu D.S., Chao N.J., Doan P.L. (2018). Epidermal growth factor and G-CSF signaling are synergistic for hematopoietic regeneration. Stem Cells.

[37] Doan P.L., Himburg H.A., Helms K., Russell J.L., Fixsen E., Quarmyne M., Harris J.R., Deoliviera D., Sullivan J.M., Chao N.J. (2013). Epidermal growth factor regulates hematopoietic regeneration following radiation injury. Nat. Med..

[38] La Cava A., Matarese G. (2004). The weight of leptin in immunity. Nat. Rev. Immunol..

[39] Francisco V., Pino J., Campos-Cabaleiro V., Ruiz-Fernández C., Mera A., Gonzalez-Gay M.A., Gómez R., Gualillo O. (2018). Obesity, fat mass and immune system: Role for leptin. Front. Physiol..

[40] Alkan Ozdemir S., Ozer E.A., Kose S., Ilhan O., Ozturk C., Sutcuoglu S. (2016). Reference values of serum IgG and IgM levels in preterm and term newborns. J. Matern. Fetal Neonatal Med..

[41] Melville J.M., Moss T.J. (2013). The immune consequences of preterm birth. Front. Neurosci..

[42] Procaccini C., Jirillo E., Matarese G. (2012). Leptin as an immunomodulator. Mol. Asp. Med..

[43] Sprockett D., Fukami T., Relman D.A. (2018). Role of priority effects in the early-life assembly of the gut microbiota. Nat. Rev. Gastroenterol. Hepatol..

[44] Dzidic M., Boix-Amorós A., Selma-Royo M., Mira A., Collado M. (2018). Gut microbiota and mucosal immunity in the neonate. Med. Sci..

[45] Correa-Rocha R., Pérez A., Lorente R., Ferrando-Martínez S., Leal M., Gurbindo D., Muñoz-Fernández M.Á. (2012). Preterm neonates show marked leukopenia and lymphopenia that are associated with increased regulatory T-cell values and diminished IL-7. Pediatrics Res..

[46] Duijts L., Bakker-Jonges L.E., Labout J.A., Jaddoe V.W., Hofman A., Steegers E.A., van Dongen J.J., Hooijkaas H., Moll H.A. (2009). Fetal growth influences lymphocyte subset counts at birth: The generation R study. Neonatology.

[47] Le Dréan G., Haure-mirande V., Ferrier L., Bonnet C., Hulin P., de Coppet P., Segain J.P. (2014). Visceral adipose tissue and leptin increase colonic epithelial tight junction permeability via a RhoA-ROCK-dependent pathway. FASEB J..

[48] Tang X., Liu H., Yang S., Li Z., Zhong J., Fang R. (2016). Epidermal Growth Factor and Intestinal Barrier Function. Mediat. Inflamm..

[49] Clark J.A., Doelle S.M., Halpern M.D., Saunders T.A., Holubec H., Dvorak K., Boitano S.A., Dvorak B. (2006). Intestinal barrier failure during experimental necrotizing enterocolitis: Protective effect of EGF treatment. Am. J. Physiol. Liver Physiol..

[50] Birchenough G.M., Johansson M.E., Gustafsson J.K., Bergström J.H., Hansson G.C. (2015). New developments in goblet cell mucus secretion and function. Mucosal Immunol..

[51] Rodewald R., Kraehenbuhl J.P. (1984). Receptor-mediated transport of IgG. J. Cell Biol..

[52] Arévalo Sureda E., Weström B., Pierzynowski S., Prykhodko O. (2016). Maturation of the intestinal epithelial barrier in neonatal rats coincides with decreased FcRn expression, replacement of vacuolated enterocytes and changed Blimp-1 expression. PLoS ONE.

[53] Kim C.Y., Kim K.H. (2014). Curcumin prevents leptin-induced tight junction dysfunction in intestinal Caco-2 BBe cells. J. Nutr. Biochem..

[54] Xu S., Wang D., Zhang P., Lin Y., Fang Z., Che L., Wu D. (2015). Oral administration of Lactococcus lactis-expressed recombinant porcine epidermal growth factor stimulates the development and promotes the health of small intestines in early-weaned piglets. J. Appl. Microbiol..

[55] Suzuki T. (2013). Regulation of intestinal epithelial permeability by tight junctions. Cell. Mol. Life Sci..

[56] Takehara M., Nishimura T., Mima S., Hoshino T., Mizushima T. (2009). Effect of claudin expression on paracellular permeability, migration and invasion of colonic cancer cells. Biol. Pharm. Bull..

[57] Bergmann K.R., Liu S.X., Tian R., Kushnir A., Turner J.R., Li H.L., Chou P.M., Weber C.R., De Plaen I.G. (2013). Bifidobacteria stabilize claudins at tight junctions and prevent intestinal barrier dysfunction in mouse necrotizing enterocolitis. Am. J. Pathol..

